# The Roles of Statistics in Human Neuroscience

**DOI:** 10.3390/brainsci9080194

**Published:** 2019-08-08

**Authors:** Oliver Y. Chén

**Affiliations:** 1 Institute of Biomedical Engineering, University of Oxford, Oxford OX1 3PJ, UK; yibing.chen@seh.ox.ac.uk; 2 Department of Psychology, Yale University, New Haven, CT 06510, USA; 3 Laboratory of Neurobiology, University College London, London WC1E 6BT, UK

**Keywords:** statistical neuroscience, brain connectivity, neural information flow, big brain data, feature selection, large-scale data decomposition, predictive modeling, brain decoding

## Abstract

Statistics plays three important roles in brain studies. They are (1) the study of differences between brains in distinctive populations; (2) the study of the variability in the structure and functioning of the brain; and (3) the study of data reduction on large-scale brain data. I discuss these concepts using examples from past and ongoing research in brain connectivity, brain information flow, information extraction from large-scale neuroimaging data, and neural predictive modeling. Having dispensed with the past, I attempt to present a few areas where statistical science facilitates brain decoding and to write prospectively, in the light of present knowledge and in the quest for artificial intelligence, about questions that statistical and neurobiological communities could work closely together to address in the future.

## 1. Prologue

What are the rules that govern the functions and the organization of the human brain? How does the nervous system give rise to sensation, cognition, and action? What are the causes of mental illnesses? 

Ever since neurology became a distinct discipline 150 years ago, neurobiologists have been seeking to answer these questions about the brain. Statisticians have played important roles in this effort, charting the brain’s functional network and topographic organization, and inventing analytical tools that pave the way for predicting brain diseases, cognition, and behaviour. Thanks to the rapid development of neuroimaging technology and the advancement of high-performance computing (HPC), an unprecedented amount of fine brain data has been collected. Using this “big brain data”, statisticians and neurobiologists are re-visiting old problems and exploring new ideas on a broader canvas. In parallel, while continuing to employ statistical methods to support modeling and data analysis in neurobiological studies, statisticians are beginning to incorporate more facts of the nervous system into developing biologically plausible frameworks.

In this brief review, I discuss recent developments in a small number of areas where statistical science has helped to advance human neuroscience. I write it partly to satisfy my curiosity and partly for the enjoyment of my friends, colleagues, and readers who are interested in studying the brain using statistical techniques. First, I discuss the impact of statistics on human neuroscience using Ronald Fisher’s three roles of statistics as a guide: the study of populations, the analysis of variance, and data reduction. Expanding on these basic concepts, I discuss four topics in brain studies in which statistical devices have been widely used: brain connectivity (the links between brain areas), information flow within and between brain regions, information extraction from large-scale neuroimaging data, and neural predictive modeling. I end by summarizing a few exciting new areas where statistics is at the heart of quantitative enquiry and in one’s quest to *decode the brain* and writing prospectively about questions and challenges with which statistics and neuroscience may deal in the future. If reading these views provides you with new insights about statistical science and brain function, then I have been amply rewarded. If some of these views stimulate new ideas and further discussions in other areas of statistical and neurobiological studies, then I have received the utmost reward.

## 2. A Statistical Enquiry Concerning the Human Brain

### 2.1. The Three Roles of Statistics in Brain Science

Ronald Fisher, the father of modern statistics, defined three concepts, which have had a profound influence in statistical and biological research: the study of populations, the analysis of variance, and the principles of data reduction [1]. During the past century, many statistical theories and methods have revolved around these themes. Today, they are helping us to analyze large-scale neural, environmental, genetic, and behavioural data, to understand the neurological bases of variability in structure and functioning of the cerebral cortex, as well as the genetic and environmental underpinnings of human behaviour.

#### 2.1.1. Three Types of Population: Hierarchies in the Human Brain

There are, broadly, three types of population in human neuroscience: the population that consists of data obtained from multiple individuals, the population that consists of data obtained from multiple time points, and the population that consists of data obtained from multiple brain areas. 

#### 2.1.2. An Example

The neural coupling (or connectivity) between brain regions outlines the anatomical and functional organization of the brain [2]. One way to quantify the strength of the coupling between brain regions is to use the Pearson correlation of the signals obtained from the two regions. This can be done by taking the correlation between two time courses measured by a neuroimaging modality, such as positron emission tomography (PET), the blood oxygen level dependent functional magnetic resonance imaging (BOLD fMRI), electroencephalogram (EEG), magnetoencephalography (MEG), or diffusion-weighted magnetic resonance imaging (diffusion MRI). The resulting correlation is called an edge.

**Figure 1:**
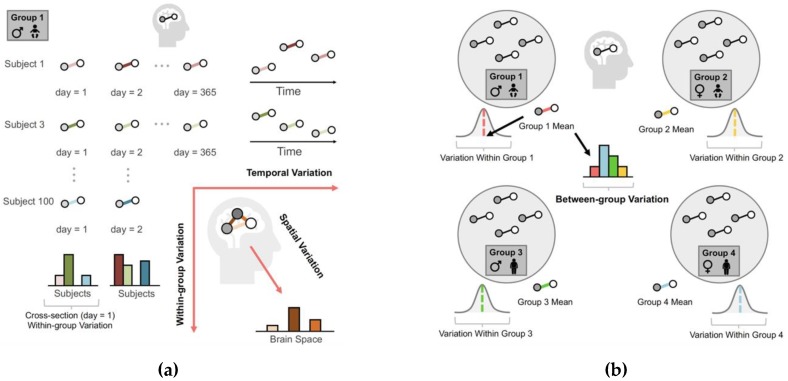
Three types of population and variation in human neuroscience. (**a**) Variation within a group. For a group of male toddlers, the three types of variation are defined according to time, (brain) space, and individuals. From left to right: temporal variation. From top to bottom: within-group variation. Bottom right: spatial variation. Note that for temporal and within-group variations, one edge is considered, while for spatial variation, multiple edges are considered since the latter considers variability across brain regions. For demonstration purposes, we show the spatial variation for one subject. (**b**) Variation between groups. We consider the variation of an edge between two brain regions in four groups (male toddlers, female toddlers, male adults, and female adults). The between-group variation quantifies the variance of group mean edge strength (coloured edges in the figure).

Let us use, as an example, one hundred individuals whose brains are scanned in an MRI scanner for one hour on New Year’s Day. The first type of population is an aggregate of edges from multiple subjects. In the first column of Figure 1a, the 100 correlations form a population of edges corresponding to 100 subjects. The second type of population is an aggregate of edges in a single brain measured over time. For example, consider one individual whose brain is scanned for one hour every day over a year. The resulting 365 correlations form a population of edges that fluctuate longitudinally (see the first row of Figure 1a). The third type of population is an aggregate of edges corresponding to multiple brain regions. The resulting region-wise correlations form a population of edges that differ from one brain area to another (see the lower right corner of Figure 1a).

#### 2.1.3. Three Classes of Variance: The Spell of Neural Diversity

Each type of population yields a unique class of variance. First, for a specific edge at a cross-section, the edge strength varies across individuals in a group, introducing the within-group variation (Figure 1a). When several heterogeneous groups are concerned, the variation from one group to another is called between-group variation (see the center image of Figure 1b). Second, an edge may change over time, exhibiting the temporal variation (Figure 1a). Finally, for an individual at a given period of time, the edge strength varies across anatomical regions, displaying the spatial variation (Figure 1a).

A central role statistical science plays in brain studies is the analysis of variance. First, comparing between-group variation with the within-group variation (of the edges), one can test the heterogeneity of edge strength across various groups. Perhaps one of the simplest examples is the one-way ANOVA *F*-test statistic, which is defined as the ratio of between-group variance over within-group variance: hence the larger the *F*-test statistic, the edges are more dissimilar between-group relative to within-group. Such a comparison also lays the foundation for the intraclass correlation (ICC): the larger an ICC, the edges are more similar within-group as compared to between-group. Additionally, the within- and between- group variations form the bases for brain data decomposition, where data derived from various experimental conditions and heterogeneous populations can be separated into population-specific components that envisage biological attributes shared by the whole population, and subject- or group-specific components that distinguish a subject or a sub-group (see Section 2.1.4) [3,4]. Second, the spatial variation, observed either at rest or under a task such as seeing a picture, highlights the areas of the brain that are more active during rest (e.g., see [5]) or are associated with the task (e.g., see [6]). Third, if the edge strength between brain areas co-varies with a behavioural or cognitive trait (e.g., fluid intelligence or cognitive ability), they are potentially modulating this trait (e.g., see [7,8]). Meanwhile, abnormal variability observed in focal brain regions suggests potential brain illness: a chief element in brain disorder diagnosis (e.g., see [9,10]). Finally, tracing temporal variation in health and disease helps to monitor brain development and brain disorder progress (e.g., see [11,12]).

#### 2.1.4. Reduction of Large-Scale Brain Data: The Local Guide to a Global Picture

Anyone studying brain data today cannot fail to be impressed by its size (dozens of petabytes or hundreds of terabytes after conversion and pre-processing). Larger-scale data contain more (although potentially noisier) information, provide a bigger platform to make scientific enquiries, and, properly treated, may produce more consistent conclusions. But there are questions to be addressed, questions that are at the interface of technology and neurobiology. Technically, a direct enquiry into the entire data set (considering mapping the dynamics of every neuron as an extreme example) may at present pose analytical and computational difficulties to methodologies and even the high-performance computers. Can we summarize the data into one with which both statistical models and computers can efficiently handle? Neurobiologically, the brain has the unique ability to extract certain constant and unchanging knowledge (such as color, orientation, and motion) from the everchanging information reaching it from the outside world. The brain is able to discard varying information and derive universal assents (such as in color vision, and generally in sensation) that allow humans (and animals) to maintain their independence from the vicissitudes of constant environmental change. As realizations of brain activities, neural data must in some way store common, perhaps genetically determined and hard-wired properties of the brain across individuals, in healthy subjects, or among patients with brain diseases. Can we uncover these common and potentially predetermined anatomical and functional properties of the brain? Living in an environment of continual change, the brain must learn to adapt and modify itself and its beliefs, enabled by its plasticity and result in variabilities. Consequently, the brain presents a certain amount of plasticity and variability in its structure and functioning. Can we derive such modifiable properties which may assist us in understanding the construction and functional organization of the brain and in deriving idiosyncratic signatures of an individual brain useful for personalized medicine and mobile health-based diagnosis?

Logically, and more realistically, can we extract a few smaller sets of “pontifical” data, that (a) summarize patterns of specific brain regions, across individuals, and of a specific subject, (b) distinguish patients from healthy subjects, and (c) potentially trace the course of mental states and the development of neurodegenerative diseases over time?

**Figure 2:**
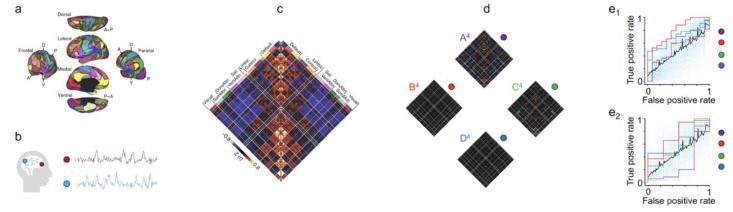
Using predictive models to uncover distinctive brain states and areas to diagnose psychosis. (**a**) The functional network organization of the human cerebral cortex is revealed through intrinsic functional connectivity. Colours reflect regions estimated to be within the same network determined based on the 17-network solution from Yeo et al. [17]. The map is displayed for multiple views of the left hemisphere in Caret PALS space [18]. (**b**) A schematic representation of time courses obtained from two brain regions. (**c**) Correlation matrices (a measurement of functional connectivity) are computed from time courses across regions. Values reflect z-transformed Pearson correlations between every region and every other region. DorsAttn indicates dorsal attention, Sal salience, SomMot somatomotor, and VentAttn ventral attention. (**d**) Brain states exhibit hierarchical features across distributed networks. Correlation matrices for four states (arranged using k-means clustering) are shown for each region. (**e**) Specific brain states are predictive of distinct clinical symptoms in novel individuals. (**e_1_**) State B^4^ uniquely predicted the presence of active psychosis in patients with psychotic illness. Results from a leave-one-subject-out cross-validation elastic-net logistic regression analysis comparing predicted and observed psychotic symptoms (*n* = 91). The displayed area under the receiver-operating characteristic curve (AUC) reflects the scalar probability measure for each individual, predicting the likelihood of active psychotic symptoms. The blue semi-transparent lines reflect 1000 ROC curves from 1000 permutation experiments. The black dashed line indicates the mean of these 1000 curves, approximating a null curve. The purple, red, green, and orange curves correspond to states A^4^, B^4^, C^4^, and D^4^, respectively. (**e_2_**) Connectivity models defined on training data predict the presence of active psychotic symptoms in an independent group of participants (*n* = 31). The displayed AUC graph reflects the scalar probability measure, predicting the likelihood of active psychosis, defined using positive edges from the initial training set without further fitting or modification, in a held-out sample of patients. The images are adapted and modified, under the terms of the Creative Commons CC BY license, from Springer Nature Communications “The human cortex possesses a reconfigurable dynamic network architecture that is disrupted in psychosis” by Reinen et al. Copyright 2018 [9].

Empirically, converging anatomical and physiological evidence suggests that the brain retains, and processes condensed and specialized information leveraging its intricate and specific structure and functioning. For example, the brain consists of functionally specialized areas [13]. It also has clearly-defined anatomical organizations, delivered by its cytoarchitecture (e.g., there is a sharp distinction between V1 and its neighbouring visual “association” cortex) [14] (p. 29), depicted in layers (for example, the lateral geniculate nucleus (LGN) consist of multiple layers each of which receives information from one eye or the other *only*) [14] (p. 26), and painted in sections (for example, the primary visual cortex is appropriately arranged in alternating bands (“L, R, L, R”) which respectively receive information from the left (L) and right (R) eyes) [15]. Finally, there exist strong correlations in large-scale brain data (for example, patterns from many of the brain areas in Figure 2c are significantly correlated [9]). Practically, brain data is contaminated with noise (for example, in fMRI data, multiples sources of noise can corrupt the true signals, such as scanner-related noise including thermal noise and scanner instability noise, noise due to head motion and physiology, HRF model errors, and noise due to different sites) [16]. It is thus necessary to reduce the amount of noise and to extract meaningful (e.g., with high signal-to-noise ratio) information from the data. Reflecting on neurobiological evidence and the practical need, it seems reasonable and useful to construct a dense subset of the brain data that summarizes properties of the whole. To rigorously execute this, we need statistical data-reduction techniques.

Data reduction is, in essence, information extraction. Two classes of data-reduction techniques, predictive-modeling based feature selection and data decomposition, are useful to treat large, multivariate brain data measured over a long time. The former works with respect to a particular predicted variable, and the latter finds general structures within the data.

**Figure 3:**
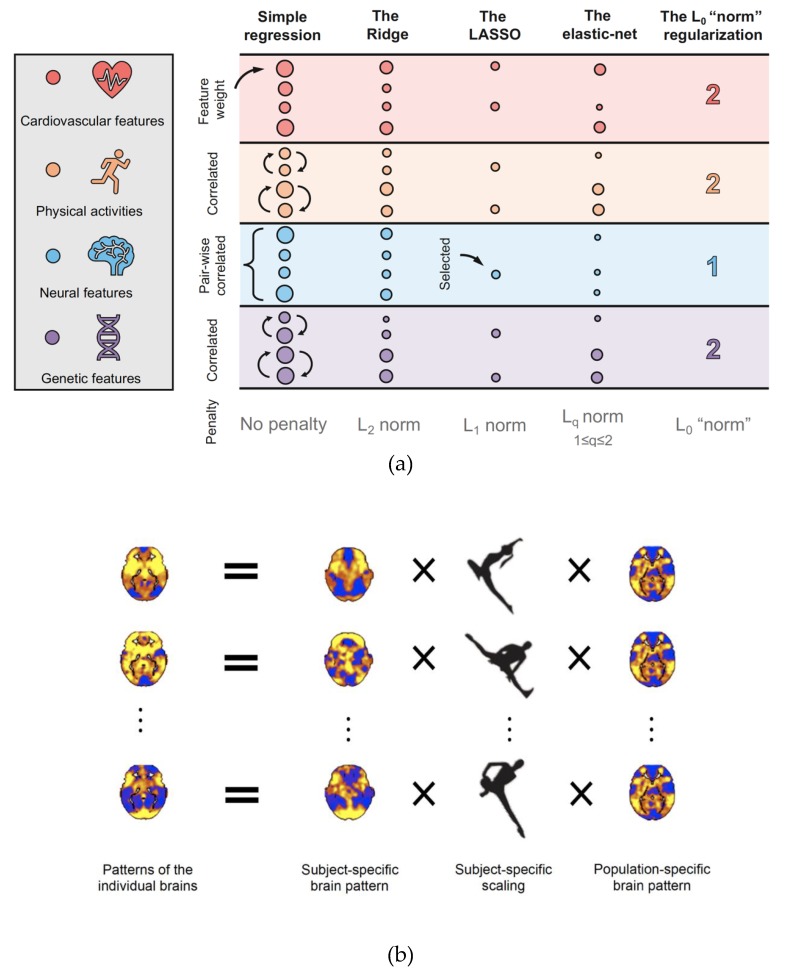
Schematic representations of data reduction on large-scale multivariate features via regularization and decomposition. (**a**) Using regularization techniques to select features. Four common regularization approaches, the Ridge, the LASSO, the elastic-net, and the L_0_ “norm” regularization, are present to analyse large-scale multivariate features from an hypothetical experiment. For demonstration purpose, consider candidate features measuring cardiovascular (such as electrocardiogram (ECG) data), behavioural (such as physical activity measurements), neural (such as fMRI data), and genetic (such as GWAS data) activities. Each colour (e.g., blue) represents multivariate features (e.g., signals obtained from different brain regions) from one feature modality (e.g., fMRI data). Real world large-scale (particularly high-dimensional) data would typically contain several clusters of features that are highly correlated within their respective clusters relative to between clusters (namely the pairwise correlations are very high within each cluster). In this demonstration, for simplicity, we assume one (e.g., for fMRI data) to two (e.g., for cardiovascular data) clusters of highly correlated features within each modality. Each circle corresponds to one feature, and the size of a circle represents the weight (estimated parameter from the model) of the feature. Assuming features are standardized, those with larger weights are more “important” features (important in terms of predicting brain disease or behavioural outputs); those with weights close to zero are discarded. The Ridge regularization (also called the L_2_ regularization), based on the L_2_ norm, imposes weights on all features but reduces the size of the weights in order to produce a stable model. It is computationally efficient, has a unique solution, but is not ideal for feature selection. The LASSO regularization (also called the L_1_ regularization), based on the L_1_ norm, shrinks the weights of some towards zero, thereby removing these features. It is computationally less efficient than the Ridge (particularly when the solution is non-sparse), has potentially multiple solutions, but is useful for feature selection. Additionally, when there are clusters of highly correlated features in data sets with more features than sample size (namely p≫n), it tends to select one feature from each cluster (see Section 2.3 in [21]). The elastic-net regularization combines the LASSO and the Ridge regularizations. It selects a small number of features (like the LASSO), some of which are correlated (like the Ridge). It is useful when one wants to balance between feature reduction and model interpretability; in other words, it attempts to reduce the number of features but does not reduce too much (as in the LASSO), so there remain a reasonable number of features for scientific interpretation. The L_0_ “norm” regularization induces sparsest solution by counting the number of non-zeros features (L_0_ is not a proper norm, hence the quotation marks). The discarded features in the LASSO have weights possibly not strictly zero (although very close to zero); in the L_0_ “norm” regularization, these features are (strictly) discarded. It is, however, computationally very expensive (NP-hard). (**b**) Using data decomposition to unveil population- and subject- specific information of the brain. Large-scale brain data obtained from multiple subjects typically encode population-specific information (which depicts the similarities across individuals) and subject-specific information (which highlights idiosyncratic patterns). Statistical data decomposition techniques, such as singular value decomposition, population value decomposition, and principal component analysis, are useful to uncover the unobserved population- and subject- specific information. For example, using the generalized population value decomposition (gPVD), the totally variation of brain patterns can be partitioned into a part that is subject-specific, a part that quantifies the subject-specific variability, and a part that is shared by a group of subjects [4].

The predictive-modeling based feature selection can be further classified into two types of models. The former type consists of step-wise models that link, at each step, one feature (e.g., an edge) with an outcome variable (e.g., disease status); judging the strength of the association, the model discards (or admits) the feature. The latter type consists of predictive models in which all features are considered simultaneously; the features whose parameter estimates from the model are not significant are discarded. Regularized models, such as the Ridge, the LASSO, and the elastic-net (see Figure 3a), which shrink the estimated parameters of irrelevant features (and thus suggest removing these features in the model output), are examples of the second type [19,20,21]. The validity of the selected features is subsequently tested on novel subjects to check if they can, without further modeling, reproduce a good outcome prediction (see Figure 2d,e).

Data decomposition considers statistical techniques that partition a large dataset into multiple smaller parts, each may shed distinctive biological lights. For example, the generalized population value decomposition (gPVD) decomposes patterns of the brain into a subject-specific part and a population-specific part (see Figure 3b) [3,4]. After carefully considering the amount of variance explained and the proper signal-to-noise ratio, the subject-specific part is useful for individual disease diagnosis and the population-specific part uncovers shared group information. Other useful data decomposition techniques include principal component analysis (PCA), whose basis functions are sorted by their ability to explain the variability of the data, independent component analysis (ICA), whose basis functions form independent subcomponents of the data, and canonical correlation analysis (CCA), which finds linear combinations between two sources of data (e.g., a set of data containing neural signals and another set containing behavior measurements) which have maximum correlation with each other (see [22] for a review).

The decomposed features can be further reduced or pruned via prediction. Oftentimes this step is necessary; this is in part because the decomposition is justified only if the reduced data sheds neurobiological light (otherwise one can propose a decomposition method that arbitrarily breaks the data apart). In other words, one needs to demonstrate that the reduced data (e.g., the subject-specific features in gPVD) is associated with a biological outcome or a stimulus. Partial least square (PLS) analysis, which simultaneously conducts dimension reduction and considers the relationship between the independent variables (e.g., neural features) and the dependent variable (e.g., behavior outcome) [23] and functional PCA (fPCA) regression, which assesses the relationship between extracted features (namely, functional principal component (fPC) scores) and the dependent variable (e.g., hypertension) [24] are useful data decomposition methods based predictive models.

### 2.2. Diagnosing Brain Diseases and Monitoring Disease Development Using Statistical Predictive Models

The hierarchical architecture of populations, the analysis of variance, and the data-reduction techniques provide conceptual and analytical tools to reach one of the crucial goals in human neuroscience research. That is, to diagnose, monitor, and, ultimately, treat brain illnesses.

**Figure 4:**
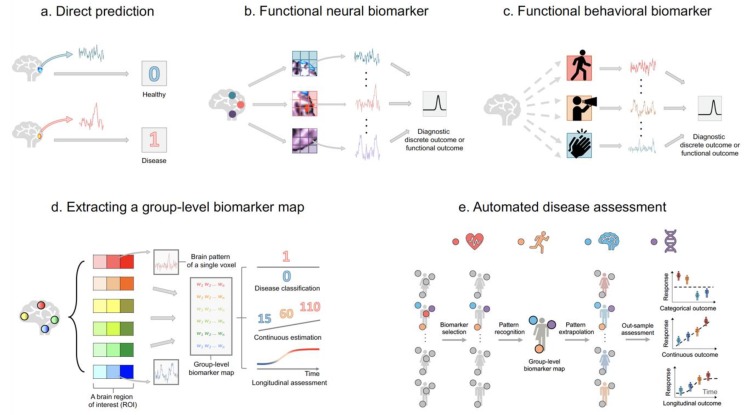
Using predictive models to diagnose disease, estimate disease severity, and assess disease progress. (**a**) A neural biomarker from a specific brain region is directly mapped to a diagnostic clinical outcome. (**b**) The functional neural biomarker framework. By evaluating the spatial pattern across subjects, the framework identifies multiple biomarkers related to diagnostic clinical outcomes. (**c**) The functional behavioural biomarker framework. When brain data is not available, behavioural data (such as physical activity, speech pattern, and hand movement) recorded semi-continuously from wearable computers serve as biomarkers. (**d**) Extracting a predictive map using functional behavioural biomarkers. Each box refers to a voxel; voxels with the same colour but different hues indicate the same anatomical or functional region. The coloured $w_{1},\;w_{2},\cdots w_{n}$ represent predictive weights for biomarkers. (**e**) Algorithm-based automated disease assessment. During model-building, features related to the heart (such as electrocardiogram (ECG) data), behaviours (such as physical activity measurements), the brain (such as fMRI data), and the genome (such as GWAS data) that are relevant to the outcome are selected. Subsequently, a predictive map consisting of weights across selected functional biomarkers is developed using the training sample. During the prospective testing step, the efficacy of selected features is verified by integrating the weight map with data from previously unseen individuals without further model fitting and yield a prediction for each subject. At a cross-section, the model yields one predicted outcome (binary disease status or continuous disease severity) per subject. Longitudinally, the model yields one predicted outcome per subject at every time point.

The simplest diagnostic model involves linking a single neural biomarker (e.g., activation from a single brain area) with a clinical outcome (e.g., healthy or diseased brain) (see Figure 4a). With the advent of functional neuroimaging data, the model extends to examine the relationship between patterns from multiple brain regions and produce a clinical outcome (see Figure 4b). As an example, linking functional connectivity features and disease status reveals disrupted neural coupling among patients with psychotic disorders. Clustering analysis on resting-state brain data demonstrates that there exist several transient connectivity-states conserved across individuals (see Figure 2d). Associating each mental state with the individual’s disease profile using a predictive model shows that one of the network states (State B in Figure 2d) is significantly disrupted in psychotic patients. This is further verified by predicting the presence of psychosis in previously unstudied individuals using the identified State B (see Figure 2e_1_,e_2_ for training and testing) [9].

Currently, scanning healthy individuals’ brain frequently in an MR machine for disease prevention is inconvenient and costly. One way to circumvent this issue is to use behavioural features measured using wearable computers (e.g., smart watches) and smartphones to assess brain illnesses (e.g., see [25] and Figure 4c). The brain is an expert in performing functional integration, where information from various functionally specialized areas (for example, area V3 is specialized in motion, V4 color, and V5 motion) are combined to form a unified sensation (such as vision). In the arena of statistics, data fusion has the potential to integrate information from multiple data sources, including cardiovascular (such as electrocardiogram (ECG) data), behavioural (such as physical activity measurements), neural (such as fMRI data), and genetic (such as GWAS data) signatures, to generate more consistent and accurate disease diagnosis (see an hypothetical experiment in Figure 4e). The journey has already begun, with exciting beginnings made by integrating fMRI and EEG data, generating higher spatial resolution images while, simultaneously, tracing the brain’s neural processes and information pathways at finer temporal scales (e.g., see [26]).

The predictive modeling-based brain disease diagnosis is not without debates, especially in the terrain of causal inference. For example, sensory inputs and cognition may be jointly associated with or mediated by “consciousness”. If irregular signals from a brain area is found to be associated with the onset of a mental disorder, one may drive into the treacherous road by concluding that signals from that area are neural markers causing the mental disorder and using them to explain mental problems. It remains possible, even adjusted for sampling bias, that the irregular signals are effects (but not causes) or that the association is due to a *spurious correlation* (in other words, some variables in a large number of features are correlated by chance). Relatedly, when the brain data are high-dimensional, they are likely to suffer from *incidental endogeneity* (where some features are coincidentally correlated with the error term (or the residual term)), thereby violating a vital model assumption. 

To deal with spurious correlation and to avoid overfitting, one could consider out-of-sample prediction, cross-validation (e.g., leave-k-subject-out cross-validation), repeated sampling test (e.g., bootstrap and permutation tests), test-retest (after out-of-sample prediction, one may conduct an additional test using data from a different study), meta-analysis (by pooling findings from multiple studies), and causal alternation (such as deep brain stimulation). The treatment for incidental endogeneity, unfortunately, is as-of-yet not well-charted. Statisticians and econometricians, however, are actively working on it and have provided inspiring and intriguing perspectives into the problem [27].

To return to the main point, despite challenges, predictive modeling has the promise to (i) identify neural and behavioural features that are associated with diagnostic clinical outcomes; (ii) assess brain diseases and their severity; (iii) monitor brain diseases over time (see Figure 4).

### 2.3. Information Flow in the Brain: A Statistical Pursuit 

Living in an everchanging world, the brain is able to modify itself to entertain the environment that is in a state of continual change. But, to what extent the wiring of the nerves is plastic and modifiable, and are modifiable neural apparatus (such as the plastic neural wiring) responsible for the way in which humans interact with the world?

**Figure 5:**
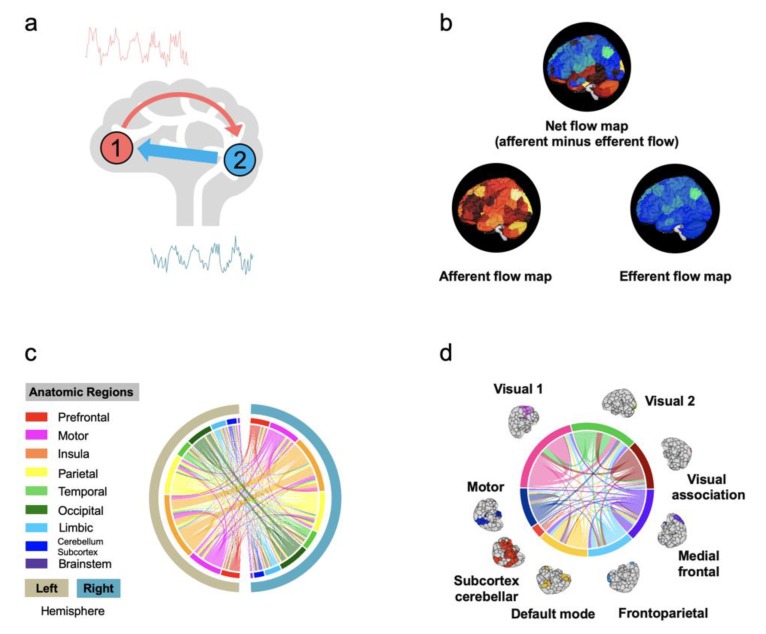
Information flows in the human brain. (**a**) Estimating information flows from a brain area 1 to another area 2—and from area 2 back to 1. This can be done using a statistical framework such as Granger causality analysis, dynamic causal modeling, and information theory (see Section 2.3). The direction and size of the arrows indicate the directed route and the amount of neural information traveling from one brain region to another. (**b**) Afferent, efferent, and net information flow maps of the whole brain. The afferent flow measures the amount of information entering a particular brain area from other brain areas. The efferent flow measures the amount of information existing from a particular brain area to other brain areas. The net information flow quantifies the difference between the two. (**c**) Average information flow between 18 anatomic regions. The 18 regions are organized based on a 268-node functional atlas. Average information flow is defined as the total amount of information flow from each anatomic region divided by the number of nodes in the region. The colour of the edge indicates the origin of the flow. For example, the pink curved line crossing the circle starting from the lower left (pink bar) to the top right (light orange bar) indicates information flow from left motor region to right insula region. (**d**) Average information flow between eight functional networks. The edges are now visualized on eight functional brain networks. The images are adapted and modified, under the terms of the Creative Commons CC BY license, from Scientific Reports “Resting-state brain information flow predicts cognitive flexibility in humans” by Chén et al. Copyright 2019 [8].

To answer the first question, a beginning can be made by enquiring into the “effective” connectivity of the brain. The study of “effective” connectivity (or directed neural information flows) (see Figure 5) is a natural extension of the study of brain connectivity (such as in Figure 2c). It uses statistical frameworks, such as Granger causality analysis [28], dynamic causal modeling [29], information theory [30], probability propagation methods (e.g., a Bayesian network [31] and pairwise conditional probability [32]), and the dynamic directional model [33], on functional imaging data to quantify the strength and direction of neural communication between paired areas and to infer potential causal relationships between these regions (see [34] for a review on methods and [35] for a review on effective connectivity). For example, studies using data from magnetoencephalography (MEG) [36] have shown that brain information flow during rest is not random but follows a posterior-to-anterior flow in high frequency bands and an anterior-to-posterior flow in low frequency bands. Data from electroencephalography (EEG) [37,38] and functional magnetic resonance imaging (fMRI) [39,40] have demonstrated that the information flow in the brain is associated with the embedded network topology.

To answer the second question, namely are modifiable neural apparatus (for example, the directed information flow) responsible for the way in which humans interact with the world, one may start with quantifying and mapping the amount of “information” that is transmitted from a brain area 1 to another area 2—and from area 2 back to 1, and subsequently, charting how this directed flow associates with human behaviour. Statistics plays an important role in this effort. First, one way to estimate the amount of information between two brain areas is to use an information metric, which represents directed edge strength, estimated from a statistical model (such as the *F* values in Granger–Geweke causal analysis, or Shannon entropy in information theory). For example, the *F* values derived from Granger causality analysis on resting-state fMRI data unveil that neural information flow profiles in the general population primarily involve local exchanges within specialized functional systems, long-distance exchanges from the dorsal brain to the ventral brain, and top-down exchanges from the higher-order systems to the primary systems (see Figure 5). Afterwards, a predictive model can be implemented to assess whether directed neural communication underpins human behavior. For example, linking the directed information flow with cognitive flexibility scores suggests that the former is significantly associated with and predictive of the latter in humans [8].

The studies of brain information flow, nevertheless, have never ceased to confront challenges. Although statistical (association) analyses have demonstrated significant links between directed and undirected brain information flows and behavioral outputs, whether the relationships are *causal* is more difficult to prove. First, despite the fact that brain imaging data possess strong neural bases (as they are highly correlated with local field potential, which is a direct measure of synaptic activity), the dynamic brain data may be confounded by metabolism and vasculature. Thus, even though one generally agrees that brain activities, in one way or another, give rise to human behaviors, it is still premature, if not erroneous, to endorse a causal claim that it is the estimated information flow that induces human behaviors, as the relationship may be confounded (by metabolism and vasculature). Second, the brain activities are measured at different time scales (for example, MEG resolves events with a precision of 10 milliseconds or faster, whereas fMRI typically resolves events with a precision of several hundred milliseconds to several seconds). Information flows estimated using different neuroimaging modalities may therefore be different (and thus need to be further validated). The sampling rate for fMRI data is slower than the timescale of the underlying neuronal responses; lag-based approaches using fMRI data, thus, require scrutiny, and need to be further verified using other data acquisition techniques, such as EEG and MEG [34,41]. 

These challenges, however, should not stop one from making causal probes into the dynamic brain. Maxwell wrote “Thoroughly conscious ignorance ... is a prelude to every real advance in knowledge.” Each statistical refinement of models and new neurobiological experiment, in my view, would offer novel insights and bring us one step closer to uncover the causal veils of dynamic neural communication.

## 3. Epilogue: The Paths to Decode the Brain?

Through the usage of neuroimaging techniques, we are beginning to uncover the neurobiological bases for sensory, perceptual, emotional, mental, and cognitive representation of the brain. Rather than detecting changes in the responses from a single cell, neuroimaging experiments measure changes in activities recorded from a large (or entire) area of the brain, when a specific task is conducted. 

**Figure 6:**
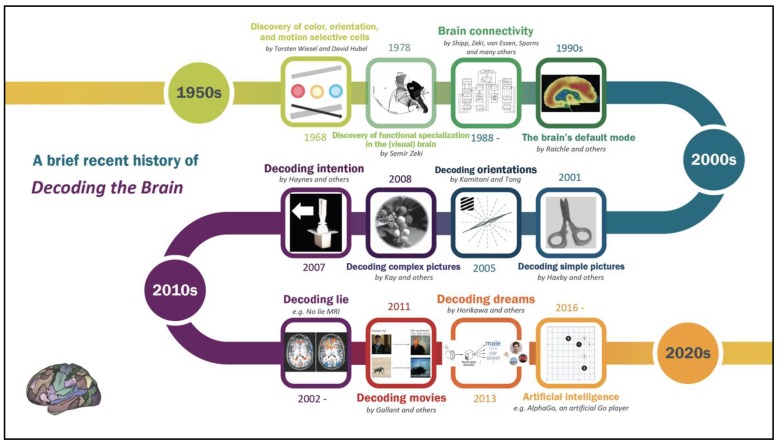
A timeline representing a few important discoveries in brain decoding during the past half-century [2,5,6,42,44,46,47,49,50,51,52].

Statistics comes firmly into the domain of brain decoding (see Figure 6). Statistical predictive models can uncover the visual stimuli viewed (e.g., an image of a pair of scissors or directed lines) based on patterns of the brain [6,42]. Brain decoding models are not restricted to simple object recognition. Using novel decoding models (e.g., a Bayesian decoder [43]), researchers are beginning to decipher brain constructs related to intention [44,45], dreams [46], and dynamic visual stimuli such as movies [47]. Statistics also plays a role in understanding representation beyond decoding models; it has helped to draw how sensory stimuli, motor responses, and cognitive processes are represented in brain patterns [48].

Moving forward, connecting statistical models, computer science technologies, and brain studies can further advance the decoding of the brain and make brain-like computers. First, there are similarities between the two, for example the anatomical demonstration of parallel connections in the visual cortex vs parallel computing and the deep neural network (DNN) and the asynchronous brain operations vs the asynchronous computer operations. Second, statistical computing and machine learning have learned remarkable lessons from experimental neurobiology. For example, inspired by the simple and complex cells in the visual cortex, Fukushima designed the Neocognitron, a hierarchical network consisting of many layers of cells, with variable connections between the cells in adjoining layers, for visual pattern recognition. Like the simple and complex cells in the visual cortex, cells in the Neocognitron are also divided into two classes: the S-cells and C-cells: the S-cells are feature-extracting cells and C-cells are inserted in the network to allow for positional errors in the features of the stimulus; each C-cell receives signals from a group of S-cells which extract the same feature, but from slightly different positions [53]. Since its invention, the Neocognitron has been used for handwritten (e.g., numerals) and pattern recognitions and laid the foundation for future convolutional neural networks (CNNs). Third, partly inspired by the organization of the nervous system, AI computers are beginning to outperform human brains in some areas. For example, industrial robots from KUKA and FANUC are today more efficient than humans in certain goal-oriented tasks such as arranging goods, palletizing, and boxing jars; IBM Watson defeated human participants during the quiz show *Jeopardy!*; more recently, AlphaGo beat professional Go players [52]. AI computers have also the potential to create unprecedented moves (strategies) that human brains have never before considered. For example, the Move 37 during Game Two of AlphaGo vs Lee Sedol was seemingly a mistake at first sight to professional human players but a well-calculated strategy retrospectively. In this sense, artificial intelligence may offer insights into the constraints of human intelligence (and perhaps decision-making), and the strategies created by machine are beginning to enter the domain of standard human strategies (such as the Move 37). Additionally, statistical simulation approaches may direct us into seas less sailed before, to enquire into questions related to one’s self, such as the questions of morality and greed, where specific experimental designs and data collection may be difficult. Inspired by human intelligence, AI has begun to benefit humanity in component tasks such as speech recognition, image classification, and autonomous vehicles; intimidatingly although speculatively, it may one day develop into a superintelligence that may not comply with human values and rules. Naturally, this raises the questions of optimizing, managing and controlling AI, some of which are being actively discussed, debated, and pursued, and to which statisticians, computer scientists, neurobiologists, and philosophers could make some contribution [54,55]: is it acceptable to design a self-driven car that treats potential accidents as probabilistic events (for example, choosing hitting two pedestrians to save four passengers in a car from falling off a cliff is probabilistically “less costly” than killing four passengers over two pedestrians; but is it moral?); how could statistical penalties and stopping rules be imposed to safeguard a goal-based AI program (which, given a goal, would make plans and exhaust resources whether adversary to humans or not until the goal is realized) once it has been launched; how could we prevent someone from taking advantage of immoral and unethical strategies AI simulated; and, more broadly, how could we avoid losing control of AI systems?

To return to nearer problems, there still seems a disconnection between statisticians and neurobiologists. The questions that “statistical neuroscientists” seek to address seem to be too vague for neurobiologists whose concerns are to understand how the real brain undertakes tasks, and many of the state-of-the-art computing frameworks (e.g., the fully-connected layers in DNN) do not reflect how the brain really works [14] (pp. 118–121). To facilitate collaborative research in the next decade in pursuing the common goal of charting the functions of the human brain and using them to benefit daily life, experimental neuroscientists need statistical models to unravel the intricacies of the brain and to learn how the nervous system undertakes complex tasks. Equally, statisticians need knowledge of the nervous system to enhance their models, ones that can be directly implemented in experimental tests to uncover how the brain may be functioning and to investigate how the brain implements defined tasks, without ignoring the underlying neurobiological, anatomical, and physiological mechanisms.

The structure and functioning of the human brain is, in part, intricately coded prenatally and, in part, diversely variable and modifiable postnatally in space, across individuals and groups, and over time. The statistical enquiry into the brain is thus a problem dissecting the intricate, universal cerebral pathways and functions which the brain uses to acquire constant knowledge (such as colour) of the external world, and the dynamic, variable, and modifiable pathways which it uses to entertain the everchanging environment. Additionally, by studying the complex pathways of the brain and its normal and abnormal functioning, one is contributing not only to an understanding of the process of knowledge, consciousness, cognition, and behaviour, but also to the perhaps more altruistic problems of understanding brain illnesses, perennial problems that are chief to clinical, public-health, and medical enquiries.

A century ago, Fisher’s introduction of population, variance, and data reduction provided fundamental statistical tools and principles that signified a beginning to understand variability in population biology under statistical frameworks. Today, they are opening new avenues for statisticians and neuroscientists to probe into the neural basis of variability in relation to human cognition, behaviour, and psychopathology. In this brief review, I have discussed how these statistical concepts have helped brain studies, including mapping the pathways between functionally and structurally coupled brain areas (the study of brain connectivity), unveiling the neural highways of the cerebral cortex (the study of brain information flow), extracting useful information from large-scale neuroimaging data (big brain data reduction), and assessing and monitoring brain illnesses (predictive neural modeling). Additionally, I presented how statistics has assisted brain decoding of objects, intentions, dreams, and movies, and how statistical and neurobiological studies could benefit each other. 

In spite of advances, the study of the brain and the field of *statistical neuroscience* are still in their infancy; many exciting ideas about them remain to be generated and to be (statistically) tested. Naturally, one cannot at present conclude all neurobiological discoveries made thus far using statistical arguments will be definitive. Naturally, too, there may be major limitations in my arguments above. There is, however, a certain degree of coherence between statistical discoveries and neurobiological hypotheses, and a great degree of confidence that there will be a stronger link between the two in the near future. The ultimate knowledge about how the brain works and how statistics can best serve neuroscience and vice versa may take many years to settle. However, no one should be deterred from asking questions, conducting experiments, and performing statistical and neurobiological analyses. It was trials and errors that established theories of colour vision, of functional specialization of the brain, and, most recently, the artificial eyes that enable self-driving cars to see.
