# Erratum: Chén “The Roles of Statistics in Human Neuroscience”, *Brain Sci.* 2019, *9*, 194

**DOI:** 10.3390/brainsci10030149

**Published:** 2020-03-04

**Authors:** Oliver Y. Chén

**Affiliations:** 1 Institute of Biomedical Engineering, University of Oxford, Oxford OX1 3PJ, UK; yibing.chen@seh.ox.ac.uk; 2 Department of Psychology, Yale University, New Haven, CT 06510, USA; 3 Laboratory of Neurobiology, University College London, London WC1E 6BT, UK

The author wishes to make an erratum to the published version of his paper [1]. In the original article, there was an error. Area V3 is specialized for dynamic form (not specialized for motion), and area V4 is specialized for color and form-with-color (not just specialized for color). A correction has been made to Section 2.2, paragraph three: “for example, area V3 is specialized for dynamic form (the shapes of objects in motion), V4 for color and form-with-color, and V5 for motion (see p. 125 in [2], p. 72 in [3], and [4]).” The author apologizes for any inconvenience this may cause to all readers.
